# Identification of α6-Containing Nicotinic Acetylcholine Receptors as the Primary Target of Nereistoxin Insecticides and Structural Basis of Channel Blockade

**DOI:** 10.3390/insects17070743

**Published:** 2026-07-21

**Authors:** Licheng Gu, Yunxin Liang, Boyan Zhang, Jia Huang, Xiaomu Qiao

**Affiliations:** 1 Collaborative Innovation Center of Green Pesticide, National Joint Local Engineering Laboratory of Biopesticide Preparation, School of Forestry and Biotechnology, Zhejiang A & F University, Hangzhou 311300, China; gulicheng@zafu.edu.cn (L.G.); yxlaign@stu.zafu.edu.cn (Y.L.); 2 Ministry of Agriculture Key Laboratory of Molecular Biology of Crop Pathogens and Insects, Institute of Insect Sciences, Zhejiang University, Hangzhou 310058, China; 3190100256@zju.edu.cn

**Keywords:** nereistoxin, molecular target, cartap, nicotinic acetylcholine receptor, *Drosophila melanogaster*, molecular docking

## Abstract

Nereistoxin (NTX) and its derivative insecticides cartap and monosultap have been used for decades to control lepidopteran pests by targeting nicotinic acetylcholine receptors (nAChRs). Unlike neonicotinoid agonists that induce excitatory neurotoxicity, nereistoxin insecticides act as antagonists, blocking cholinergic neurotransmission. However, the molecular target and mechanism of action of these compounds remain incompletely understood. Here, we elucidated the mode of action of cartap and monosultap in *Drosophila melanogaster* through integrated genetic and computational approaches. Bioassays with *Drosophila* nAChR subunit mutants demonstrated that the α6 subunit is critically required for insecticidal activity of both compounds, with α6 knockout conferring approximately 10-fold resistance to monosultap and 7-fold resistance to cartap. Molecular docking of protonated NTX into an α6 homopentameric channel model revealed a “dual-anchor” blocking mechanism: the protonated amine forms electrostatic interactions with residue Glu267, while the dithiolane ring creates steric hindrance at residue Thr270. Pore diameter measurements showed an optimal binding cavity of 5.96–6.22 Å in the 267–270 region, narrowing dramatically to 1.64 Å at the deep gate (Ser278), explaining how NTX binding physically occludes the channel. Collectively, these results identify α6-containing nAChRs as the primary target of nereistoxin insecticides and provide a structural framework for understanding channel blockade, with important implications for resistance monitoring and the development of next-generation channel-blocking insecticides.

## Simple Summary

Cartap and monosultap are widely used insecticides for controlling agricultural pests, but their precise mode of action remains uncertain. Using fruit flies as a model, we found that these insecticides target the α6-containing nicotinic acetylcholine receptor (nAChR). We also showed that the active toxin blocks the receptor channel, preventing nerve cells from communicating normally. This study explains how nereistoxin insecticides work at the molecular level and provides information that could support the development of improved insecticides and resistance-management strategies.

## 1. Introduction

The nereistoxin class of insecticides, derived from the marine annelid toxin nereistoxin (NTX) isolated from *Lumbriconereis heteropoda*, has played an important role in agricultural pest management for decades [1,2]. Cartap, a bis(thiocarbamate) derivative, and its analog monosultap are commercially successful insecticides that exhibit potent activity against lepidopteran pests, particularly rice stem borers [3,4]. Unlike neonicotinoid insecticides that act as agonists at nicotinic acetylcholine receptors (nAChRs), nereistoxin and its derivatives function as antagonists, blocking cholinergic neurotransmission and causing inhibitory neurotoxicity [4,5].

Nereistoxin analogues are proinsecticides; they are not fully active in their parent form and are converted in vivo or in the environment to NTX. Cartap is a well-known example. It undergoes rapid pH-dependent hydrolysis, generating cartap monothiol, cartap dithiol, and ultimately NTX, with dithiol being proposed as a plausible direct channel-blocking agent [6].

At the target level, studies on vertebrate nAChRs have shown that NTX displays subtype-specific binding affinities, with α4β2 neuronal receptors showing the highest sensitivity [7]. Early investigations on vertebrate preparations suggested that NTX acts primarily at the agonist binding site [8], while subsequent studies on insect receptors revealed voltage-dependent channel blockade [9], indicating potential species-specific differences in NTX action. Recent studies suggested that NTX may have dual actions at both sites, acting as a noncompetitive channel blocker as well as a weak agonist site ligand [10]. However, the precise nAChR subunit composition targeted by nereistoxin insecticides in insects, and the structural basis of channel blockade, remain incompletely understood.

In this study, we investigate the mode of action of cartap and monosultap in *Drosophila melanogaster* using genetic and computational approaches. Through bioassays with nAChR subunit knockout mutants, we identify the specific subunits required for insecticidal activity. Furthermore, molecular docking simulations elucidate the structural basis of nereistoxin binding within the ion channel pore, providing a framework for understanding channel blockade.

## 2. Materials and Methods

### 2.1. Chemicals

Cartap hydrochloride (>99%, Cat# C1433387) and monosultap (>96%, Cat# T684841) were purchased from Aladdin, Shanghai, China.

### 2.2. Fly Strains

Flies were maintained and reared on conventional cornmeal agar molasses medium at 25 ± 1 °C and 60% ± 10% humidity with a photoperiod of 12 h light:12 h night. The w^1118^ (#5905) strain was used as the wild-type control. All nAChR KO mutants were gifts from Dr. Yi Rao (Peking University). The nAChR β1^R81T^ mutant was produced in our previous study by CRISPR/Cas9 genome editing [11].

### 2.3. Insecticide Bioassays

To assess insecticide resistance in different fly strains, adult female flies aged three to five days and of uniform size were selected for bioassays. The Insecticide Resistance Action Committee (IRAC) susceptibility test method 026 (https://irac-online.org/methods/, accessed on 1 April 2025) was followed with minor modifications. Briefly, serial dilutions of monosultap or cartap solutions were prepared in 200 g/L sucrose, with approximately 5 mL required for each concentration. A 1 cm piece of dental wick was placed in a standard *Drosophila* vial and treated with 800 μL of 20% sucrose, with or without the insecticide. Ten flies of each genotype were then transferred into the vials, and each genotype was tested in at least three replicates for every insecticide concentration. To prevent flies from becoming trapped in the dental wick, the vials were kept upside down until all the flies became active. After 48 h, flies showing no movement in response to gentle prodding with a fine brush were scored as dead. For each insecticide, at least five concentrations were used with three replicates per concentration, and the entire experimental series was repeated in triplicate. LC_50_ values were determined using probit analysis with Polo Plus software version 2.0 (Leora Software, Berkeley, CA, USA). Resistance ratios (RR) for each mutant were calculated by dividing the mutant’s LC_50_ by the LC_50_ of the w^1118^ wild-type control.

### 2.4. Modeling of nAChR and Molecular Docking

To elucidate the structural basis of nereistoxin channel blockade, the α6 homopentameric configuration was selected as the structural model based on several lines of evidence. First, our previous genetic studies demonstrated that spinosyns act specifically through α6-containing nAChRs, whereas the evolutionarily related α5 and α7 double-knockout mutants remain fully susceptible to spinosyns [11]. Second, α6 has been shown to form functional homomeric channels in the presence of the ancillary protein NACHO [12]. Third, in the present study, deletion of α6 conferred by far the highest level of resistance, whereas mutations in β1 and other subunits commonly associated with heteromeric receptors had little or no effect on susceptibility. Collectively, these findings support the use of an α6 homopentamer as a parsimonious structural model for investigating the pore-blocking mechanism of nereistoxin.

The model was generated using residues 1–494 of the *D. melanogaster* α6 subunit (NCBI Reference Sequence: NP_995674.1) using the AlphaFold3 server. Model reliability was assessed using AlphaFold’s confidence metrics. The generated model yielded a predicted template modeling (pTM) score of 0.80 and an interface predicted template modeling (ipTM) score of 0.80, indicating high overall topological accuracy and highly confident subunit–subunit interface predictions. The raw AlphaFold3 model file and the full confidence metrics file can be found in the Supplementary Materials.

To investigate the structural basis of protonated nereistoxin (NTX) binding within the M2 transmembrane pore, molecular docking simulations were performed using the AutoDock Vina 1.2.0 software package. The 3D structure of protonated NTX was prepared, minimized, and assigned with Gasteiger charges using AutoDockTools. The receptor grid box was dedicatedly centered within the extracellular vestibule of the nAChR α6 homopentamer to encompass the lumen channel space flanked by residues Glu267 and Thr270. The search space dimensions were defined as 25 × 25 × 25 Å^3^ with an exhaustiveness value set to 32 to guarantee thorough conformational sampling. The binding poses were evaluated based on the affinity scoring function, and the top-ranked conformation that exhibited optimal charge and steric complementarity within the 267–270 region was selected for further pore profile characterization.

The structural dimensions and topology of the internal M2 transmembrane conduction pathway were characterized using the CAVER 3.0 software package [13] integrated with PyMOL (Version 3.1.3.1). To precisely trace the axial trajectory across the drug-binding region, a calculation starting seed point was accurately anchored within the pore lumen near the selectivity filter using specified Cartesian coordinates (X=−14.996, Y=21.151, Z=−11.487). Given the severe occlusion influx blocked by insecticide binding within the channel, a high-resolution routing strategy with a fine probe radius of 0.5 Å was intentionally deployed. This configuration was strictly required to mathematically resolve the sub-angstrom spatial bottlenecks without inducing premature path searching termination, thereby ensuring a continuous geometric profile of the pore. Local cross-sectional diameters along the central axis were automatically extracted from the calculated profiles to quantify the dimensions of the NTX-binding pocket (Glu267 and Thr270) and the downstream structural gate (Ser278). Longitudinal clipping (slab views omitting two forward subunits) and high-resolution rendering were executed in PyMOL for visualization.

## 3. Results

### 3.1. Differential Resistance Profiles of nAChR Subunit Mutants to Monosultap and Cartap

To identify the nAChR subunits essential for the insecticidal activity of nereistoxin-class insecticides, we evaluated the susceptibility of *Drosophila* nAChR subunit knockout mutants to monosultap and cartap using feeding bioassays.

For cartap, the α6 knockout mutant again demonstrated the highest resistance level, with an resistance ratio (RR) of 6.84-fold (LC_50_ = 1666.60 mg/L vs. 243.62 mg/L in control). The α1, α2, α3, and β3 mutants displayed moderate resistance with RR values ranging from 1.92 to 2.15. Notably, the α4^T227M^, α5, α7, β1^R81T^, and β2 mutants showed susceptibility levels comparable to or even greater than the wild-type control (RR ≤ 1.35), with the α5 mutant displaying significantly increased sensitivity (RR = 0.43) (Table 1).

For monosultap, the α6 subunit knockout mutant exhibited the highest level of resistance, with an LC_50_ RR of approximately 9.98-fold compared to the wild-type control (LC_50_ = 4287.56 mg/L vs. 429.55 mg/L in control). The α4^T227M^ mutant showed moderate resistance with an RR of 4.67, while α3 and β3 mutants displayed RR values of 4.06 and 4.33, respectively. The α1, α2, α7, β1^R81T^, and β2 mutants showed low but statistically significant resistance (RR ranging from 3.30 to 3.88). In contrast, the α5 mutant showed near-normal susceptibility (RR = 1.24) (Table 2).

These results reveal that the α6 subunit is critically required for the insecticidal activity of both cartap and monosultap, suggesting that nereistoxin insecticides primarily target nAChR subtypes containing the α6 subunit.

### 3.2. Molecular Docking of Protonated Nereistoxin in the nAChR Ion Channel Pore

**Figure 1:**
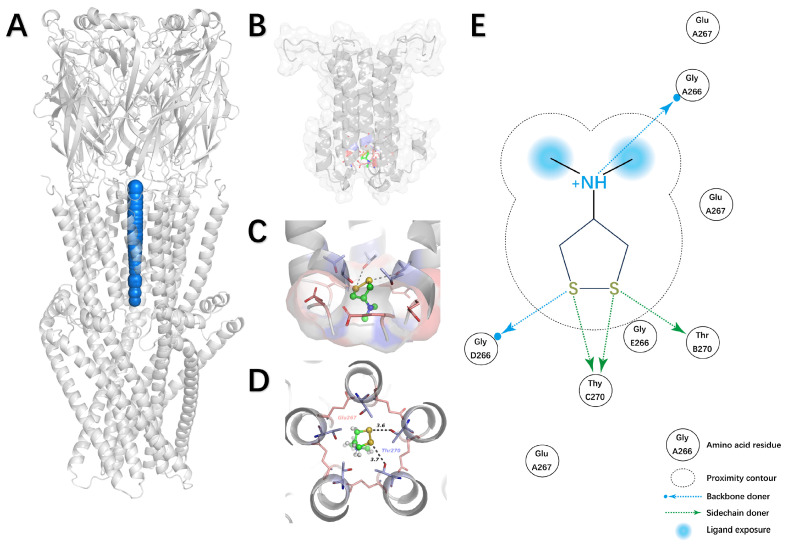
Molecular docking model of nereistoxin with the M2 receptor pore. (**A**) The α6 homopentameric nAChR is displayed as a translucent molecular surface (Grey), with the channel lumen highlighted in blue. (**B**) Close-up view of the M2 channel pore with protonated nereistoxin. (**C**) Side view, (**D**) top view and (**E**) diagram of the binding pocket. Protonated nereistoxin (Yellow sticks) is embedded in the center of the binding pocket. Key interacting residues Glu267 (Salmon) and Thr270 (light blue) are shown as thin sticks, with their side chains oriented toward the channel center forming tight contacts with the toxin.

To elucidate the structural basis of nereistoxin channel blockade, we performed molecular docking simulations of protonated NTX with the AlphaFold3-predicted structural model of the insect nAChR α6 homopentameric ion channel. The docking model suggests that protonated NTX is positioned within the lumen of the M2 transmembrane pore and supports a putative “dual-anchor” blocking mechanism, in which the protonated amine is predicted to be stabilized by electrostatic interaction with Glu267, while the dithiolane ring is accommodated within the narrow pore region adjacent to Thr270, resulting in simultaneous electrostatic anchoring and steric confinement (Figure 1A,B).

The binding interaction is primarily driven by strong charge complementarity between the positively charged protonated amine group of NTX and polar residues lining the channel wall. The docking model suggests that the protonated amine of NTX may interact electrostatically with the side chain of Glu267 (salmon), whereas the dithiolane ring is positioned adjacent to Thr270 (light blue), where it may contribute steric confinement within the narrow pore (Figure 1C,D). Based on the docking model, we propose that this putative dual-anchor mechanism may explain how protonated NTX occludes the ion-conducting pathway (Figure 1E)

Channel pore diameter calculations provided quantitative support for the proposed binding model. In the region spanning residues 267–270, where protonated NTX is predicted to bind, the pore diameter measures approximately 5.96–6.22 Å. This dimension is sufficiently wide to accommodate the NTX molecule (typical short axis of 4–7 Å) while remaining narrow enough to permit close contact between the ligand and pore-lining residues. In contrast, deeper in the channel at residue Ser278, the pore diameter narrows to approximately 1.64 Å, forming the narrowest constriction within the predicted pore. This observation is consistent with a resting- or closed-state conformation of the predicted channel model, as the constriction is substantially narrower than the kinetic diameter of a hydrated ion or even a water molecule (~2.8 Å). Within this structural framework, docking suggests that NTX occupying the 267–270 region may physically occlude the upper pore and thereby impede ion permeation by restricting access to the deeper constriction. Together, the pore geometry illustrates the funnel-like architecture of the channel and provides a plausible structural explanation for how NTX may block ion conduction.

The comparison of these pore dimensions supports a compelling structural interpretation: protonated NTX is predicted to bind within the 267–270 region, where the pore diameter measures approximately 5.96–6.22 Å, resulting in physical occlusion of the channel lumen. Although the Ser278 forms a narrower constriction that would itself restrict ion flow, NTX is positioned in the more extracellular region of the pore, where it may prevent ions from accessing the deeper gate. Based on the docking model, we propose a putative “dual-anchor” mechanism in which electrostatic interaction between the protonated amine and Glu267, together with steric confinement imposed by the dithiolane ring near Thr270, may contribute to channel blockade by nereistoxin insecticides.

## 4. Discussion

The identification of nAChR subunit requirements for nereistoxin insecticide activity represents a significant advance in understanding their mode of action. Our resistance profiling across *Drosophila* nAChR subunit mutants demonstrates that the α6 subunit is critically required for the insecticidal activity of both monosultap and cartap. For monosultap, α6 knockout resulted in approximately 10-fold resistance, while for cartap, α6 deletion conferred nearly 7-fold resistance—the highest resistance ratios observed among all mutants tested for each compound (Table 1 and Table 2). These findings establish that α6-containing receptors—likely forming homopentameric channels—serve as the primary in vivo target for these compounds.

This target specificity of nereistoxin insecticides closely resembles that of spinosyns (e.g., spinosad and spinetoram). Previous studies have shown that spinosyns act almost exclusively on homopentameric receptors formed by the α6 subunit, while having little to no effect on other types of nAChRs [11]. Although spinosyns are allosteric modulators, whereas nereistoxin analogues are considered ion channel blockers, both selectively target α6 homomeric pentamers in vivo. These findings raise the possibility that naturally occurring α6 null mutations could confer cross-resistance to both two insecticide classes. However, no field evidence of target-site cross-resistance has been reported to date. On the other hand, neonicotinoids such as cycloxaprid and imidacloprid, which primarily target heteromeric subtypes composed of α1, α2, β1, and β2 subunits [11,14]. The minimal resistance observed in the β1^R81T^ mutant, which confers high-level neonicotinoid resistance [15], confirms that nereistoxin and neonicotinoid insecticides do not share target-site cross-resistance, supporting their rotational use in resistance management.

Nevertheless, moderate resistance was also observed in several other subunit mutants, including α1, α2, α3, β3, and the α4^T227M^ mutant, particularly for monosultap. Given the diversity of nAChR subtypes in insects, these subunits may participate in alternative receptor assemblies that contribute, albeit to a lesser extent, to the toxic action of nereistoxin insecticides. In addition, compensatory changes in nAChR subunit expression or receptor composition following genetic disruption could partially account for the modest resistance or, in the case of cartap on the α5 mutant, the increased susceptibility [16]. Furthermore, because monosultap and cartap are proinsecticides that require metabolic conversion to the active metabolite NTX, differences in their activation efficiency may influence the effective concentration of NTX reaching the receptor. Such pharmacokinetic differences may therefore contribute to the distinct resistance ratios observed between the two compounds.

Our molecular docking studies provide a structural model of nereistoxin interaction with the insect nAChR ion channel pore. The docking model supports a proposed “dual-anchor” blocking mechanism that provides a plausible explanation for the potency and specificity of nereistoxin insecticides. The protonated amine group forms electrostatic interactions with Glu267, anchoring the toxin in the upper channel region, while the dithiolane ring creates steric hindrance at the narrower region around Thr270. This “anchor-plug” mechanism effectively occludes the channel, preventing ion flow and blocking neurotransmission. The quantitative pore diameter measurements are consistent with this proposed binding model and provide structural support for the hypothesized channel-blocking mechanism.

It should be noted that the proposed dual-anchor mechanism is derived from static molecular docking and should therefore be regarded as a working hypothesis rather than definitive structural evidence. Although the docking model suggests that Glu267 and Thr270 may contribute to electrostatic interaction and steric confinement of protonated NTX, respectively, these proposed roles remain to be experimentally validated. Future studies combining site-directed mutagenesis, molecular dynamics simulations, and binding free-energy analyses will be valuable for evaluating the individual contributions of these residues and further refining the proposed mechanism of channel blockade.

The differential binding affinities of NTX for vertebrate nAChR subtypes [7] provide important context for understanding its mechanism of action. Notably, NTX displays very low affinity for the agonist binding site (IC_50_ > 1000 μM for [^3^H]IMI displacement), while effectively displacing [^3^H]TCP, a ligand for the channel blocker site. This pharmacological profile supports the conclusion that channel blockade—rather than competitive antagonism at the orthosteric site—is the primary mechanism of nereistoxin action, consistent with our structural data demonstrating direct pore occlusion.

In summary, this study establishes that cartap and monosultap exert their insecticidal activity by targeting α6 homopentameric nAChR in insects. This unique mode of action, characterized by a specific channel-blockade mechanism within the α6 pore, parallels the target specificity of spinosyns while remaining distinct from neonicotinoids. These insights are essential for ongoing resistance monitoring and the development of next-generation insecticides that exploit specific ion channel architectures. The RDL GABA receptor, targeted by cyclodiene organochlorines and phenylpyrazoles, demonstrates the viability of channel-blocking insecticides against ligand-gated ion channels. This precedent supports α6-containing nAChRs as similarly attractive targets. Although our structural model remains a hypothesis requiring experimental validation, it identifies key pore features that may guide future structure-based optimization of α6-targeted blockers.

## Figures and Tables

**Table 1:** Log dose probit mortality data and resistance ratios for cartap.

Strain	LC_50_	50% CL	LC_95_	95% CL	Resistance Ratio	
	(mg/L)		(mg/L)		LC_50_	LC_95_
Control	243.618	186.143–346.942	476.569	365.958–750.196	1	1
α1^-/-^	524.448	422.299–677.487	1004.598	813.086–1377.867	2.15	2.11
α2^-/-^	468.105	400.387–561.221	886.958	751.928–1110.820	1.92	1.86
α3^-/-^	493.304	372.904–674.749	1073.653	840.516–1566.149	2.02	2.25
α4^T227M/-^	212.908	142.400–307.589	572.334	435.664–886.936	0.87	1.2
α5^-/-^	104.482	83.562–130.221	243.533	200.384–324.153	0.43	0.51
α6^-/-^	1666.6	1421.223–2002.049	3395.353	2884.668–4190.555	6.84	7.12
α7^-/-^	248.739	208.347–305.080	523.125	436.775–668.336	1.02	1.1
β1^R81T^	328.827	274.261–399.566	647.874	547.713–808.546	1.35	1.36
β2^-/-^	228.372	119.040–458.281	488.406	332.621–1286.236	0.94	1.02
β3^-/-^	271.45	219.637–346.085	525.206	427.956–713.422	1.11	1.1

**Table 2:** Log dose probit mortality data and resistance ratios for monosultap.

Strain	LC_50_	50% CL	LC_95_	95% CL	Resistance Ratio	
	(mg/L)		(mg/L)		LC_50_	LC_95_
Control	429.55	370.08–507.81	799.25	687.03–972.93	1	1
α1^-/-^	1480.27	1202.10–1850.27	4075.16	3364.60–5267.61	3.45	5.1
α2^-/-^	1668.27	1388.19–2029.30	4383.71	3684.23–5505.88	3.88	5.48
α3^-/-^	1743.43	1389.73–2157.38	4983.89	4179.76–6281.34	4.06	6.24
α4^T227M^/^-^	2006.37	1696.25–2376.50	5721.97	4928.26–6895.07	4.67	7.16
α5^-/-^	532.19	416.26–662.72	1409.06	1174.86–1806.40	1.24	1.76
α6^-/-^	4287.56	3813.57–4884.66	8490.45	7471.05–9969.59	9.98	10.62
α7^-/-^	1418.81	1204.48–1679.25	3387.07	2913.32–4102.28	3.3	4.24
β1^R81T^	1432.1	1001.00–1925.48	4915.62	3958.89–6629.23	3.33	6.15
β2^-/-^	1574.67	1393.15–1791.80	3302.91	2928.90–3819.23	3.67	4.13
β3^-/-^	1857.82	1622.98–2150.18	4219.55	3678.70–5004.04	4.33	5.28

## Data Availability

The data presented in this study are available on request from the corresponding authors.

## Supplementary Materials

The following supporting information can be downloaded at: https://www.mdpi.com/article/10.3390/insects17070743/s1.
